# Evolutionary loss of 8-oxo-G repair components among eukaryotes

**DOI:** 10.1186/2041-9414-1-12

**Published:** 2010-09-01

**Authors:** Kristina Jansson, Anders Blomberg, Per Sunnerhagen, Magnus Alm Rosenblad

**Affiliations:** 1 Department of Cell and Molecular Biology, Lundberg Laboratory, University of Gothenburg, P.O. Box 462, S-405 30 Göteborg, Sweden

## Abstract

**Background:**

We have examined the phylogenetic pattern among eukaryotes of homologues of the *E. coli *7,8-dihydro-8-oxoguanine (8-oxo-G) repair enzymes MutY, MutM, and MutT.

**Results:**

These DNA repair enzymes are present in all large phylogenetic groups, with MutM homologues being the most universally conserved. All chordates and echinoderms were found to possess all three 8-oxo-G repair components. Likewise, the red and green algae examined have all three repair enzymes, while all land-living plants have MutY and MutM homologues, but lack MutT. However, for some phyla, *e.g. *protostomes, a more patchy distribution was found. Nematodes provide a striking example, where *Caenorhabditis *is the only identified example of an organism group having none of the three repair enzymes, while the genome of another nematode, *Trichinella spiralis*, instead encodes all three. The most complex distribution exists in fungi, where many different patterns of retention or loss of the three repair components are found. In addition, we found sequence insertions near or within the catalytic sites of MutY, MutM, and MutT to be present in some subgroups of Ascomycetes.

**Conclusion:**

The 8-oxo-G repair enzymes are ancient in origin, and loss of individual 8-oxo-G repair components at several distinct points in evolution appears to be the most likely explanation for the phylogenetic pattern among eukaryotes.

## Background

To maintain structural integrity of DNA, organisms have developed DNA repair mechanisms. These have evolved both in complexity and specificity to ensure genomic integrity against the constant threats from damaging agents of endogenous and exogenous origins. Damage to DNA bases resulting from alkylation, oxidation, deamination, and UV-induced crosslinking, is mainly repaired by the base excision repair (BER) pathway, which is highly conserved throughout evolution and ubiquitously present in bacteria, archaea, and eukaryotes [1]. BER is the major pathway for repair of oxidative base damage, transcription-coupled repair (TCR) and mismatch repair (MMR) being important backup pathways. Moreover, several of the DNA glycosylases that initiate BER of oxidative damage have overlapping specificities and serve as alternative pathways for various DNA lesions [2]. Oxidative damage in DNA, specifically the 8-oxo-G lesion, is removed or prevented by the 8-oxo-G-specific BER enzymes MutY, MutM, and MutT [3]. MutT is an 8-oxo-dGTPase that prevents incorporation of 8-oxo-G into DNA [4]. MutM excises 8-oxo-G paired with C [5], while MutY is an adenine-DNA glycosylase that excises A paired with 8-oxo-G [6]. The MutY and MutM glycosylases are both members of the helix-hairpin-helix (HhH) superfamily. This gene subfamily is the most diverse of the DNA glycosylases, with differing substrate specificities [7]. The MutT homologue belongs to the group of nudix hydrolases and is not classified as a DNA glycosylase, despite being a component of the 8-oxo-G repair system [1].

The existence of an 8-oxo-G repair system in all main organism groups; archaea, bacteria, fungi, animals, and plants, underscores the importance of this system to defend against deleterious 8-oxo-G mutagenesis. Despite the widespread conservation and importance of this repair system in maintaining genomic stability, limited phylogenetic data is available about the highly diverse and adaptable HhH gene family of DNA repair enzymes among eukaryotes. The expanding number of entire genome sequences from a wide range of eukaryotic groups therefore encouraged an analysis of the phylogenetic distribution of the 8-oxo-G repair genes. As a broad phylogenetic analysis of the HhH superfamily of BER DNA glycosylases among prokaryotes has already been presented [7], prokaryotes were omitted from this study. Here, we have identified 8-oxo-G repair genes from metazoans, fungi and plants, along with a sequence analysis of the identified proteins.

We find that their phylogenetic distribution among eukaryotes strongly argues for group-specific gene losses. Thus, we reveal several cases of unexpected gene distributions, despite the fact that our analysis includes organisms where DNA repair has been extensively characterised both biochemically and genetically: yeast, mammals, and higher plants.

## Results

### Phylogenetic distribution

**Figure 1:**
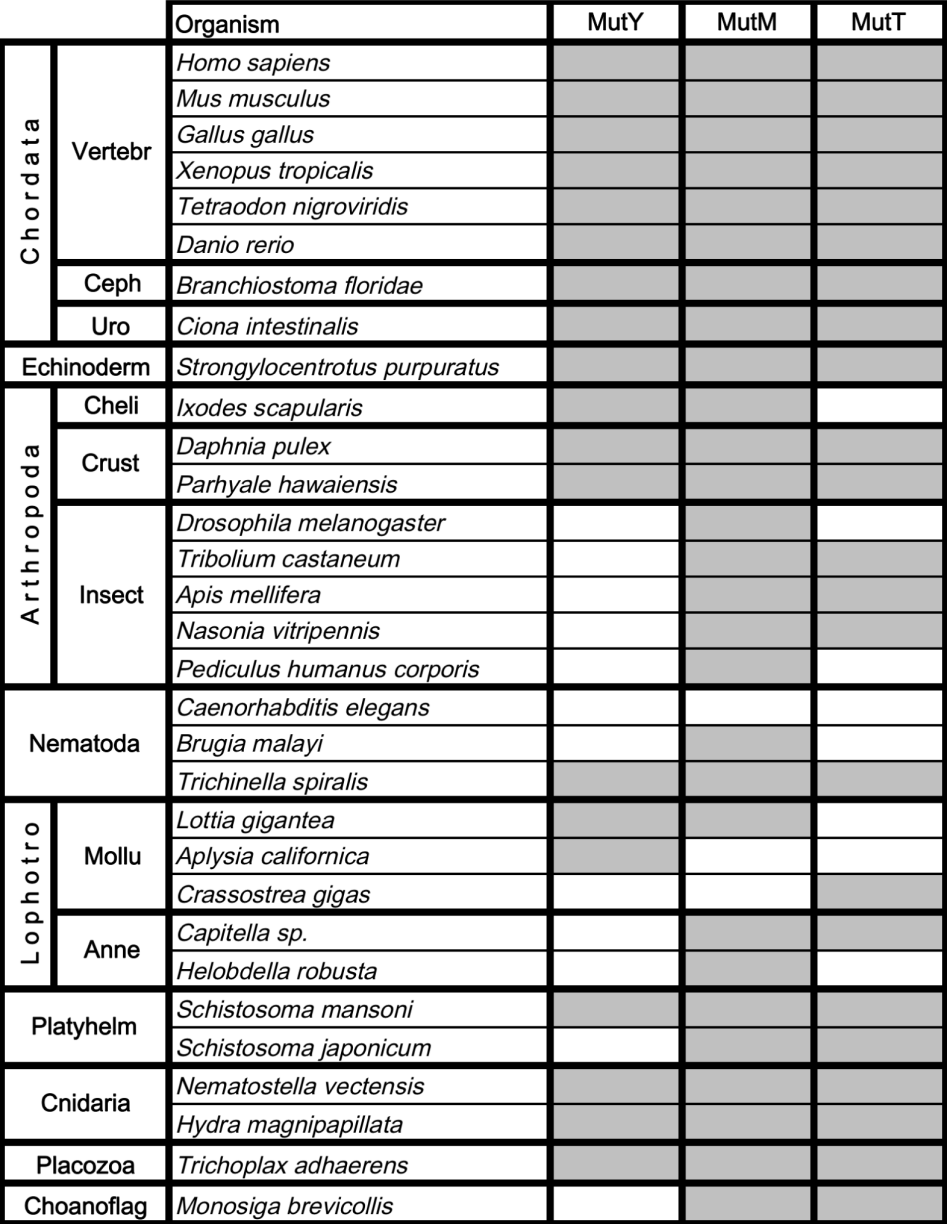
**Phylogenetic distribution of MutY, MutM, and MutT homologues among metazoans**. The table shows the presence (grey) or absence (white) of 8-oxo-G repair gene homologues found in species included in the survey. Abbreviations: "Vertebr", Vertebrata; "Ceph", Cephalochordata; "Uro", Urochordata; "Cheli", Chelicerata; "Crust", Crustacea; "Lophotro", Lophotrochozoa; "Mollu", Mollusca; "Anne", Annelida; "Platyhelm", Platyhelmintes; "Choanoflag", Choanoflagellata.

**Figure 2:**
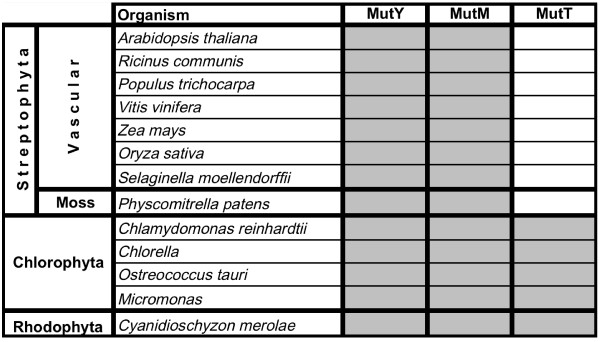
**Phylogenetic distribution of MutY, MutM, and MutT homologues in pants**. The table shows the presence (grey) or absence (white) of 8-oxo-G repair gene homologues found in species included in the survey. "Vascular", Tracheophyta.

**Figure 3:**
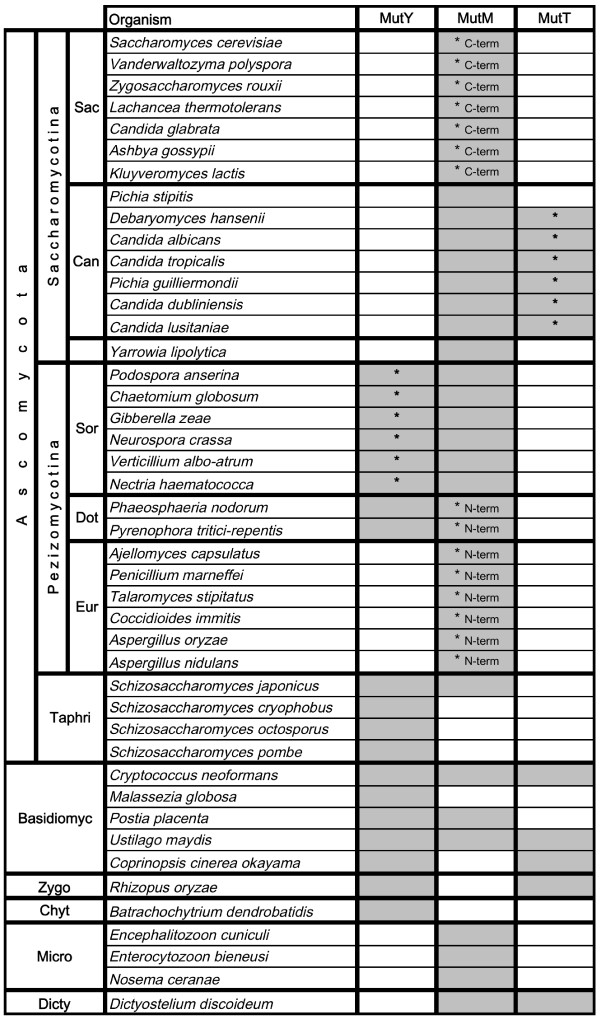
**Phylogenetic distribution of MutY, MutM, and MutT homologues among fungi**. The table shows the presence (grey) or absence (white) of 8-oxo-G repair gene homologues found in species included in the survey (fungi and slime mold). Repair enzyme homologues with any of the identified sequence inserts are marked with an asterisk, and where relevant their position indicated (C-term, N-term). "Sac", Saccharomyces; "Can", Candida; "Sor", Sordariomycetes; "Dot", Dothideomycetes; "Eur", Eurotiomycetes; "Taphri", Taphrinomycotina; "Basidiomyc", Basidiomycetes; "Zygo", Zygomycetes; "Chyt", Chytridiomycetes; "Micro", Microsporidia; "Dicty", Dictyostelia.

The phylogenetic distribution and sequence analysis of the 8-oxo-G repair components included a large number of species across the domains of fungi, animals, and plants. In general, homologues of all three types of 8-oxo-G involved enzymes MutY, MutM, and MutT were found in most surveyed subgroups of animals and plants with a few exceptions (Figures 1, 2 and 3). All three 8-oxo-G repair enzymes are found throughout the entire group of chordates (8 species examined; Fig. 1). In red and green algae, the distribution pattern is likewise uniform, with all three repair homologues found in all species examined. Notably, all eight species of land-living plants examined lack a MutT homologue, but do possess MutY and MutM homologues (Fig. 2). However, MutY homologues are apparently absent in insects and annelids (Fig. 1). Notably, the *Caenorhabditis *nematodes harbour none of the three 8-oxo-G repair genes of interest. Interestingly, all three enzymes are found in the nematode *Trichinella spiralis *and MutM is found in *Brugia malayi*, showing that nematodes have evolved several different ways to deal with 8-oxo-G damage. Each of the two nematodes *C. elegans *and *C. briggsae *harbours one HhH superfamily homologue from the Nth BER family [7], which may functionally overlap and explain the lack of the 8-oxo-G repair system. Similarly, the molluscs display a very scattered distribution, with no single species harbouring all three 8-oxo-G repair proteins. However, considering molluscs as a group, all enzyme members of the 8-oxo-G repair system are represented (Fig. 1). Incomplete genome sequencing cannot be ruled out as a source of error, however.

The situation in fungi looks more diverse. Overall, all three 8-oxo-G repair homologues are found in basidiomycetous fungi (Fig. 3). However, among ascomycotes, the phylogenetic distribution pattern is clear but patchy. Of the three proteins, MutT is the least prevalent in Ascomycota, and is retained only in the "Candida" group. All ascomycetous fungi were found to harbour the MutM homolog, with the exception of *Schizosaccharomyces*, although in this clade the single species *Sz. japonicus *seems to have the MutM protein as well. The MutY homologue is absent not only from *Saccharomyces cerevisiae *and closely related species, but also from other organisms of Saccharomycotina. Interestingly, the MutY component is found in Sordariomycetes and Dothideomycetes (subgroups of Pezizomycotina), but not in Eurotiomycetes, indicating it has been lost several times during evolution of the ascomycotes. MutY was present in all *Schizosaccharomyces *species, which was expected as *Sz. pombe *has a well-characterised MutY homologue.

Overall, the MutM homologue emerges as the most prevalent repair 8-oxo-G component among eukaryotes.

### Sequence divergence between orthologues

**Figure 4:**
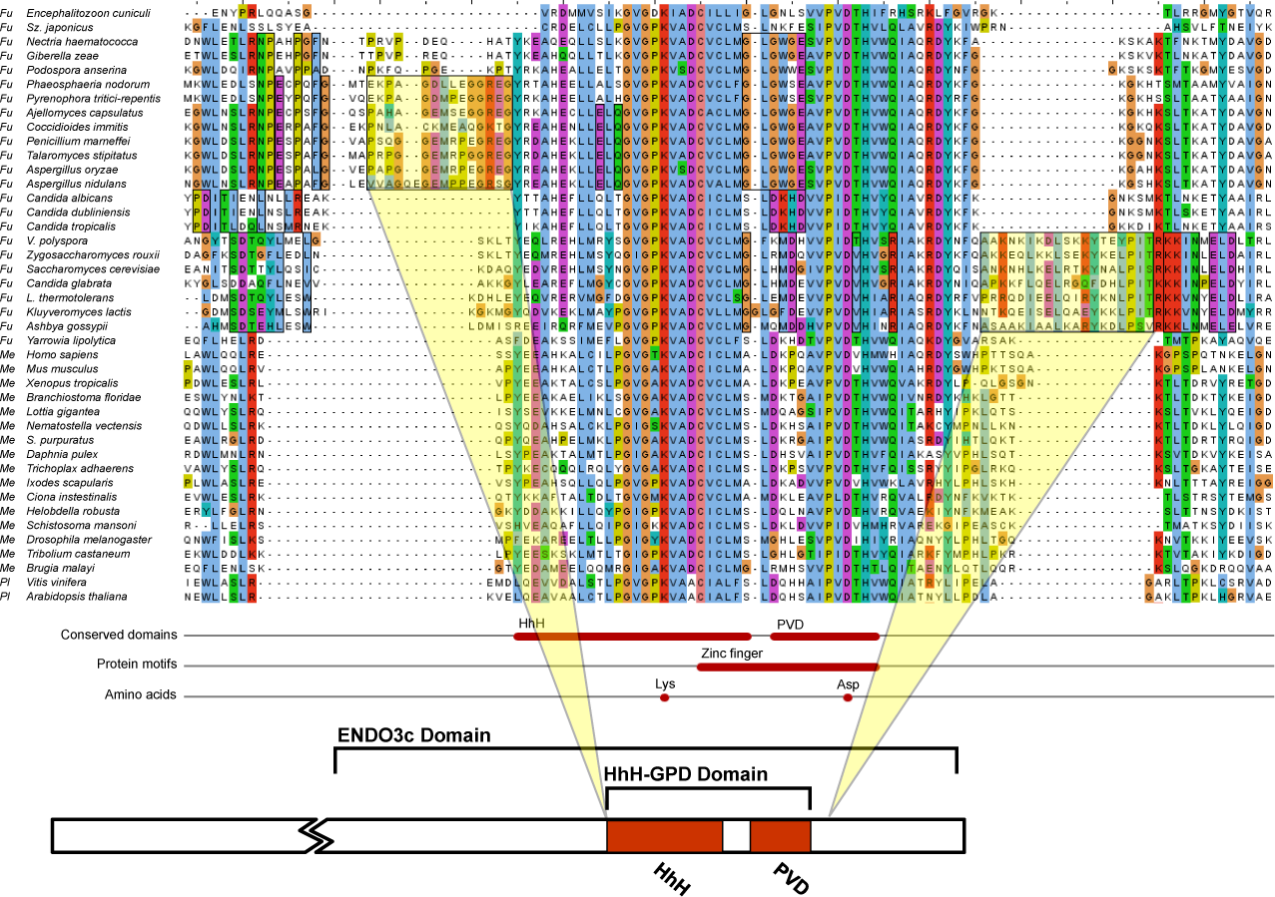
**Sequence alignment of fungal, metazoan, and plant MutMH**. The alignment shows the HhH structural domain, and the two identified MutMH sequence inserts (yellow shaded boxes) within the fungal groups Eurotiomycetes and "Saccharomyces" respectively. The Eurotiomycetes MutMH insert is located immediately upstream of the HhH domain, while the "Saccharomyces" insert resides close to the C-terminal end of the HhH domain.

**Figure 5:**
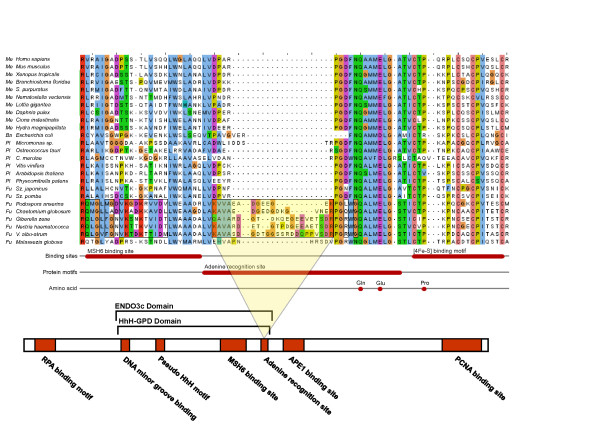
**Sequence alignment of fungal, metazoan, and plant MutYH**. The alignment shows the C-terminal end of the important HhH structural domain, and the identified MutYH sequence insert (yellow shaded box) within the fungal group Sordariomycetes. The insert is located in the MutYH substrate binding site.

To see if sequence divergence of the three repair proteins correlates with phylogeny, or would be dependent on the presence or absence of other 8-oxo-G repair components within any given organism, multiple sequence alignments were constructed (Figs 4 and 5). Among the 8-oxo-G enzymes, only the sequence of MutY is highly conserved between prokaryotes and eukaryotes [8]. MutY has a catalytic domain containing a signature helix-hairpin-helix element, followed by a Gly/Pro-rich loop and a catalytically essential aspartate residue, referred to as the HhH-GPD motif. MutY is distinct among the HhH-GPD superfamily in that it contains an additional carboxy-terminal domain that seems to be responsible for 8-oxo-G recognition [9]. The two MutY helical domains form a positively-charged groove with the adenine specific pocket at their interface. Like MutY, other base excision repair HhH superfamily glycosylases (MutM, EndoIII and AlkA) use similar two-domain molecular scaffolds, and their DNA-binding HhH-GPD motifs is the most conserved superfamily structural element [10]. In general, a strong conservation of the defined protein domains of MutY, MutM, and MutT was found. Identified residues, critical for DNA binding and substrate interaction [11-13], are extremely well conserved through all species examined. However, the group of fungi shows some unique sequence features. The "Saccharomyces" subgroup within Saccharomycotina is found to harbour an insert in MutM, approximately 15 amino acids long, about 10 residues downstream from the HhH-PVD structural domain (Fig. 4). Furthermore, the Eurotiomycetes subgroup of Pezizomycotina harbours another MutM insert, located immediately upstream of the HhH-PVD domain, and of the same size (about 10 residues; Fig. 4). Conversely, the Saccharomycotina group "Candida" and the Pezizomycotina group Sordariomycetes, that do not harbour any of the two MutM insertions, instead were found to have a small MutT sequence insertion and a longer MutY insertion, respectively (Fig. 3). The MutY insertion is located in the beginning of the specific "adenine recognition site", the substrate binding domain of the MutY protein (Fig. 5). While the longer, about 20 residues, MutY insertion is located in the important substrate recognition site, the shorter MutT insertion is located outside the highly conserved structural "nudix motif" in the catalytic site of the protein (not shown). Thus, all identified sequence insertions are found only among ascomycetous species, largely paralleling the phylogenetic division into subgroups. Interestingly, no single fungal species carries more than one out of the four identified sequence insertions (two in MutM, one in MutY, one in MutT). Construction of phylogenetic trees from entire protein sequences, in trying to clarify any mutually dependent evolutionary relationship between the 8-oxo-G repair components and these sequence insertions, did not provide any further information.

## Discussion

**Figure 6:**
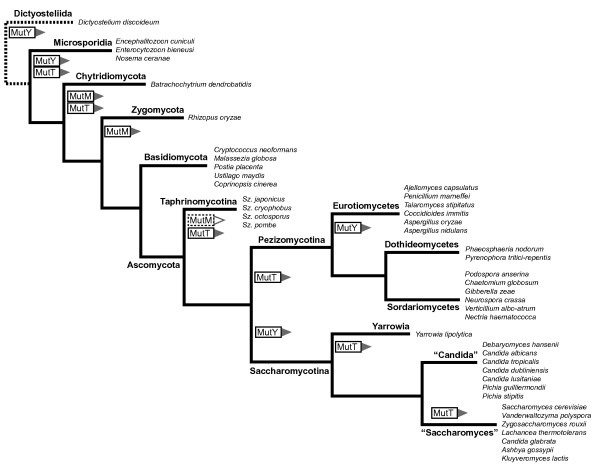
**Evolutionary loss of individual 8-oxo-G repair proteins among fungi**. Loss of a specific repair protein is indicated by the homologue name in the outlined branches. The retention of MutM in *Sz.japonicus *marked as dashed box. The basidiomycetes are the only subgroup harbouring all three 8-oxo-G repair components. The schematic tree is based on Fitzpatrick et al., 2006 [18].

Evolutionary loss of individual 8-oxo-G repair proteins is the most likely event behind the observed distribution pattern of the MutM, MutY, and MutT proteins among eukaryotes. The existence of highly conserved full-length protein sequences throughout the lineage of evolution strongly argues for group-specific gene losses, and rules out convergent evolution of independently evolved catalytic domains of repair genes among diverse subgroups of eukaryotic species. However, it is not obvious why individual 8-oxo-G repair homologues have been lost or retained in an organism group. In fungi, the loss of specific 8-oxo-G repair genes in distinct phylogenetic branches is very clear. Fungi within Pezizomycotina have lost the MutT homolog, while Saccharomycotina species seem to have lost the MutY homologue (Fig. 6). The Eurotiomycetes branch of Pezizomycotina moreover has lost the MutY protein, and harbours only the MutM homolog. The "Saccharomyces" and "Yarrowia" subgroups of Saccharomycotina also show loss of the MutT homolog, while it is still present in the "Candida" branch of Saccharomycotina (Fig. 6).

The "Saccharomyces", like the Eurotiomycetes subgroups, harbour only the MutM homolog. All subgroups of Ascomycota clearly harbour the MutM repair enzyme, except Schizosaccharomyces. Interestingly, in this clade the single species *Sz. japonicus *harbours a MutM homologue, with a sequence similar to other fungal homologues. By contrast, in *Sz. pombe*, MutT- and MutM-like proteins have not been identified either by sequence or experimental analysis [14]. Also, the nematode *C. elegans *deviates from the expected distribution pattern of 8-oxo-G repair enzymes, in that it harbours none of the 8-oxo-G repair components. However, *C. elegans *does hold the Nth HhH superfamily homologue, which may function in an alternative 8-oxo-G repair pathway [7]. It is noteworthy that *Caenorhabditis *thus only has one HhH superfamily member. By contrast, the nematode *Trichinella spiralis *harbours all three of the Mut proteins. This probably reflects the large evolutionary distance between nematode subgroups, with *T. spiralis *belonging to a basal lineage and evolutionary distant to *C. elegans*. Another noteworthy example is *Drosophila melanogaster*, which only has a MutM homologue, whereas most arthropods have two to three 8-oxo-G repair proteins. Therefore, the common picture of 8-oxo-G repair gene distribution, predicted from a typical "model organism", is not always the most representative view.

The widespread distribution of the MutM homologue in eukaryotic genomes, and the lack of either the MutY or the MutT homolog, or both, probably indicates 8-oxo-G in non-replicated DNA as the most abundant and important oxidative DNA damage to correct. The post-replicative adenine DNA glycosylase MutY mainly serves to excise adenines misincorporated opposite 8-oxo-G by replication, in cooperation with the MMR system [15]. This likely provides redundancy in post-replicative mismatch repair by separate pathways. All three 8-oxo-G repair components, however, are highly specific for their substrates [16], and possibly may have evolved from more "promiscuous" BER repair enzymes with catalytic activity toward alternative substrates. The situation of combining "promiscuous" broad substrate enzymes with highly specific ones may provide an advantage in terms of specificity and redundancy within and between separate DNA repair pathways [16]. The organism thereby holds the capacity to deal with a larger variety of DNA damage in a new complex chemical environment.

The identified sequence insertions in MutY, MutM, and MutT, respectively, among subgroups of fungi, do not obviously correlate with the presence or absence of the other 8-oxo-G repair homologues. It is also not possible to predict the functional importance of these sequence insertions for specific enzymatic activities. The sequence diversity among HhH glycosylases, and to some extent observed among subgroups of fungi in this study of the 8-oxo-G repair system, may reflect the need of specific and highly adaptable systems to process complex patterns of DNA damage, caused by different environmental factors. Disparities in catalytic mechanisms and in DNA repair pathways, by which an organism processes DNA damage, probably is part of the explanation [7]. Even though there is an established functional redundancy between the MutY, MutM, and MutT proteins [17], and between separate repair pathways, in protecting against oxidative damage, more experimental data in substrate specificity and DNA repair pathway redundancy are clearly needed.

## Methods

Eukaryotic organisms were included in the study if the entire genome sequence was available, with the aim of covering as wide a selection of organism groups as possible. Published sequences of MutY, MutM and MutT from *Schizosaccharomyces pombe*, *Saccharomyces cerevisiae*, human, and *Arabidopsis thaliana *were used in BLASTP searches (E-values < 1 × 10^-20^) to retrieve candidate homologues in the respective groups of fungi, metazoans, and plants, plus the slime mold *Dictyostelium *and the choanoflagellate *Monosiga *(for summary of data, see Additional file 1, 2, 3 and 4). Evaluation of candidates was based on the identification of domains in the NCBI Conserved Domains Database (CDD) that are specific for the different proteins: Endo3c (cd00056) and DNAglycosylase_C (cd03431) for MutY, OGG_N (pfam07934, or in some cases cl06806) and Endo3c for MutM, and MTH1 (cd03427) for MutT. The domains were identified as part of the NCBI web-based BLAST interface which includes an RPS-BLAST search vs. the position-specific scoring matrices in CDD. While E-values were different for the four domains, they were always lower than 1 × 10^-05 ^(cd03431). Only specific hits to domains were considered and best hits to similar domains (for instance Nth) were used as evidence to reject a candidate. For MutY and MutM, both domains had to be identified to score as positive. Found sequences were also subjected to reciprocal BLASTP searches, ensuring that they indeed were most similar to proteins of the respective family. Most searches were conducted using the non-redundant protein database at NCBI of December 2009.

Although the domain search made identifying proteins with low similarity to the initial query sequences easier, reliable candidates from the performed searches were also used as queries to retrieve more sequences from closely related groups, especially when a thorough evaluation was needed because the identified candidate had a different length.

As most protein sequences are based on gene predictions, many sequences had truncated ends due to the problems of identifying the exon-intron structure and thus the true ends of the gene. In those cases, the candidate protein sequence was extended by matching full sequences from closely related organisms to the genome using TBLASTN and adding the found segments to the protein sequence. For some organisms, also EST data were used to validate a predicted sequence. Long gaps or insertions within sequences that are not conserved in related species, and thus are indications of erroneous gene predictions, were left uncorrected as long as they did not interfere with the identification of domains and could be aligned properly to the other sequences.

To get a better representation of species in the different groups and conduct searches against unpublished genomes and/or EST libraries, organism-specific databases were also used, many hosted by the Joint Genome Institute (JGI; Table 1). In the summary of results (Figs 1, 2 and 3), some species in groups with many representatives are left out (*e.g. Drosophila *and *Saccharomyces *species). In a few cases, species with only EST data available were added to show that a specific protein was indeed found within the group (*e.g. *the mollusc *Crassostrea gigas*).

**Table 1:** Organism-specific databases utilised in this study.

Species	Organization	URL
*Apis mellifera*	BeeBase	http://genomes.arc.georgetown.edu/drupal/beebase/

*Batrachochytrium dendrobatidis*	Broad	http://www.broadinstitute.org/annotation/genome/batrachochytrium_dendrobatidis/

*Capitella teleta*	JGI	http://genome.jgi-psf.org/Capca1/Capca1.home.html

*Chlorella *NC64A	JGI	http://genome.jgi-psf.org/ChlNC64A_1/ChlNC64A_1.home.html

*Chlorella vulgaris*	JGI	http://genome.jgi-psf.org/Chlvu1/Chlvu1.home.html

*Ciona intestinalis*	JGI	http://genome.jgi-psf.org/Cioin2/Cioin2.home.html

*Cyanidioschyzon merolae*	Univ. of Tokyo	http://merolae.biol.s.u-tokyo.ac.jp/

*Daphnia pulex*	JGI	http://genome.jgi-psf.org/Dappu1/Dappu1.home.html

*Daphnia pulex*	wFleaBase	http://wfleabase.org/

*Dictyostelium discoideum*	dictyBase	http://www.dictybase.org/

*Helobdella robusta*	JGI	http://genome.jgi-psf.org/Helro1/Helro1.home.html

*Lottia gigantea*	JGI	http://genome.jgi-psf.org/Lotgi1/Lotgi1.home.html

*Monosiga brevicollis*	JGI	http://genome.jgi-psf.org/Monbr1/Monbr1.home.html

*Parhyale hawaiensis*	JGI	http://genome.jgi-psf.org/parha/parha.home.html

*Rhizopus oryzae*	Broad	http://www.broadinstitute.org/annotation/genome/rhizopus_oryzae/

*Schizosaccharomyces octosporus*	Broad	http://www.broadinstitute.org/annotation/genome/schizosaccharomyces_group/

*Selaginella moellendorffii*	JGI	http://genome.jgi-psf.org/Selmo1/Selmo1.home.html

*Stagonospora *(*Phaeosphaeria*) *nodorum*	Broad	http://www.broadinstitute.org/annotation/genome/stagonospora_nodorum.2/

*Trichoplax adhaerens*	JGI	http://genome.jgi-psf.org/Triad1/Triad1.home.html

*Xenopus (Silurana) tropicalis*	JGI	http://genome.jgi-psf.org/Xentr4/Xentr4.home.html

Multiple alignments were constructed using clustalw or t_coffee and visualised in JalView where also annotated features, such as the MutY "adenine recognition site", were included (Figs 1 and 2). The locations of found insertions were mapped to known 3D structures of the proteins from the Protein Data Bank, accessed via NCBI Structure database, using the program Cn3D. Structures used in the analysis were: MutY from *Geobacillus stearothermophilus*, PDB:1RRQ; MutM from *Homo sapiens*, PDB:2NOF; MutT from *E. coli*, PDB:3A6S.

## Competing interests

The authors declare that they have no competing interests.

## Authors' contributions

PS conceived of the study and drafted the manuscript. MAR devised the design of the study and carried out some of the searches and gene predictions, as well as analyses of the results. KJ performed the majority of the searches and sequence alignments, put together the results and helped draft the manuscript. AB helped conceive the study and revised the manuscript. All authors read and approved the final manuscript.

## Supplementary Material

Additional file 1 **Sequence Data**. Excel file with information on all sequences included in study and data from searches. Sheets for Fungi, Metazoa and Plants plus overview for each group. Table contains accession numbers for MutY/M/T orthologous sequences as well as information on BLASTP hit values and domain hits. Bullets symbolize reliable hits for a species to a certain query and may be found in two columns for each Mut protein: "MutX" or "MutX additional refs" when additional query sequences from closely related species have been used. The initially used query sequences are listed in row 2. Comments, such as truncated domains, are listed in "Note" column. Click here for file

Additional file 2 **MutY alignment**. For MutY and MutM most sequences from tables are included. For MutT only fungi and human sequences are included. All sequences are named with a code that identifies the organism group: 'Me' = metazoa; 'Fu' = fungi; 'Pl' = plants; 'Ba' = bacteria. Gaps in all sequences are the result of insertions in sequences removed from alignment. Annotations from literature are added. Secondary structure was taken from predictions using the "PHYRE automatic fold recognition server" (http://www.sbg.bio.ic.ac.uk/phyre/). Regions that are judged as different in a group of organisms are marked with boxes. Click here for file

Additional file 3 **MutM alignment**. For MutY and MutM most sequences from tables are included. For MutT only fungi and human sequences are included. All sequences are named with a code that identifies the organism group: 'Me' = metazoa; 'Fu' = fungi; 'Pl' = plants; 'Ba' = bacteria. Gaps in all sequences are the result of insertions in sequences removed from alignment. Annotations from literature are added. Secondary structure was taken from predictions using the "PHYRE automatic fold recognition server" (http://www.sbg.bio.ic.ac.uk/phyre/). Regions that are judged as different in a group of organisms are marked with boxes. Click here for file

Additional file 4 **MutT alignment**. For MutY and MutM most sequences from tables are included. For MutT only fungi and human sequences are included. All sequences are named with a code that identifies the organism group: 'Me' = metazoa; 'Fu' = fungi; 'Pl' = plants; 'Ba' = bacteria. Gaps in all sequences are the result of insertions in sequences removed from alignment. Annotations from literature are added. Secondary structure was taken from predictions using the "PHYRE automatic fold recognition server" (http://www.sbg.bio.ic.ac.uk/phyre/). Regions that are judged as different in a group of organisms are marked with boxes. Click here for file
