# Supplementary figures and images for: Evolutionary loss of 8-oxo-G repair components among eukaryotes
Source: Genome Integr. 2010 Sep 1;1:12. doi: 10.1186/2041-9414-1-12 (PMC2941479; doi:10.1186/2041-9414-1-12)

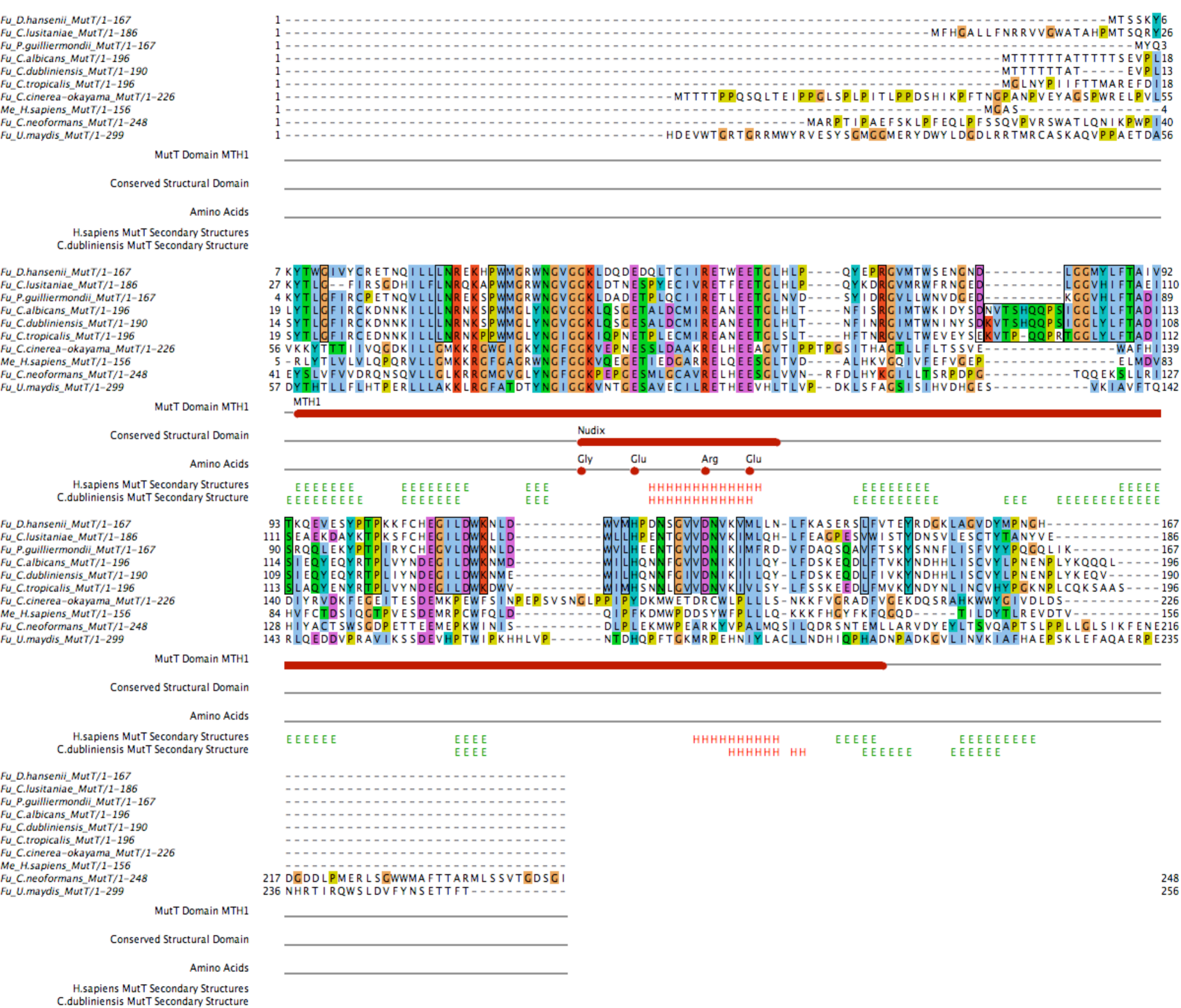

Supplement: Additional file 4 — MutT alignment. For MutY and MutM most sequences from tables are included. For MutT only fungi and human sequences are included. All sequences are named with a code that identifies the organism group: 'Me' = metazoa; 'Fu' = fungi; 'Pl' = plants; 'Ba' = bacteria. Gaps in all sequences are the result of insertions in sequences removed from alignment. Annotations from literature are added. Secondary structure was taken from predictions using the "PHYRE automatic fold recognition server" (http://www.sbg.bio.ic.ac.uk/phyre/). Regions that are judged as different in a group of organisms are marked with boxes. [file 2041-9414-1-12-S4.PDF]
